# On the Frequent Occurrence of Alkaline Urine in Health, &c.

**Published:** 1848-02

**Authors:** 


8. *On the frequent occurrence of Alkaline Urine in Health, and the Errors of Diagnosis, occasioned by a want of knowledge of this fact.* By Dr. Krukenberg.

The fact, first promulgated by Wohler, that the internal use of salts of vegetable acids and fruits containing them, causes the urine to be secreted alkaline, has been too much neglected by succeeding physiologists and pathologists. Our author found that a much smaller quantity of fruit was necessary for the production of this phenomenon than has hitherto been supposed, viz.: 2 to 4 oz. of apple pulp, or 12 plums, weighing without the stones scarcely 1½ oz., sufficed to make the urine alkaline and hazy from phosphates, or if clear on excretion, heat caused their deposition; the addition of a little hydrochloric acid caused an efferverscence like champagne; too much liquid, a bladder already filled with acid urine, or a disproportionate allowance of flesh, interfered with the succsss of the experiment. How often are those ill of chronic complaints who use a moderate diet, and with whom fruit is a useful and favorite article, troubled with hazy and alkaline urine, causing anxiety alike to themselves and their physician, which a little physiology does away with. In the simple chronic nephritis of Rayer, the chief symptom is the alkalinity of the urine; in no case was there a sectio cadaveris; and some of the cases recovered so quickly, as to justify a doubt as to the correctness of the diagnosis; although he inculcates careful dietetic treatment, it is evident from his work that the semiotic influence of fruit in small quantities was unknown to him. This article is not forbidden at La Charité, and friends of the patients often carry them some. In several of his cases the alkalinity of the urine seemed to depend on purulent admixture, and consequent rapid putrefaction; and in one it seemed to be kept up, if not produced, by the use of an alkaline saline water (Contrexeville), The alkalinity of the urine has also been used by Prout as a diagnostic sign of certain spinal affections. These he divides into two great classes:—1st. Those arising from depressing emotions and weakening influences; and in these he recommends the use of fruit, and fluids containing malic acid, as cider and perry; to these, and not to any disease, our author refers the alkalinity of the urine. 2d. Injuries of the spine; our author states, that neither Rayer nor himself had ever been able to observe the urine alkaline in cases of injuries of the spine, unless there were some existing or consecutive affection of the mucous membrane of the urinary passages, producing purulent admixture, hastening thereby the putrefactive changes in the urine. In the three cases detailed by Prout, two had stricture of the urethra, and the third retraction of the testicle, and a mucous sediment—all bespeaking the existence of some such affection. A microscopical examination, by showing the existence or absence of pus-cells in the urine, would have confirmed the diagnosis, or at once corrected it. How far inattention to diet may have led to error, cannot be specified. Prout also mentions, without explanation, what has already been referred to, viz.: That although alkaline urine, by copious secretion, be clear and bright, yet boiling causes it to deposite a phosphatic sediment, which falls without any such previous process, if the secretion be more sparing; the phosphates separate before the boiling point, and from their great specific gravity fall rapidly, and may thereby, as well as by their solubility in acids, be distinguished from the albumen found in Bright's disease.—*Month. Jour., Aug.* 1847. *From ibid.*

